# Symptoms of cybersex addiction can be linked to both approaching and avoiding pornographic stimuli: results from an analog sample of regular cybersex users

**DOI:** 10.3389/fpsyg.2015.00653

**Published:** 2015-05-22

**Authors:** Jan Snagowski, Matthias Brand

**Affiliations:** ^1^Department of General Psychology: Cognition, University of Duisburg-EssenDuisburg, Germany; ^2^Erwin L. Hahn Institute for Magnetic Resonance ImagingEssen, Germany

**Keywords:** cybersex addiction, sexual arousal, problematic sexual behavior, approach avoidance, behavioral addictions

## Abstract

There is no consensus regarding the phenomenology, classification, and diagnostic criteria of cybersex addiction. Some approaches point toward similarities to substance dependencies for which approach/avoidance tendencies are crucial mechanisms. Several researchers have argued that within an addiction-related decision situation, individuals might either show tendencies to approach or avoid addiction-related stimuli. In the current study 123 heterosexual males completed an Approach-Avoidance-Task (AAT; Rinck and Becker, 2007) modified with pornographic pictures. During the AAT participants either had to push pornographic stimuli away or pull them toward themselves with a joystick. Sensitivity toward sexual excitation, problematic sexual behavior, and tendencies toward cybersex addiction were assessed with questionnaires. Results showed that individuals with tendencies toward cybersex addiction tended to either approach or avoid pornographic stimuli. Additionally, moderated regression analyses revealed that individuals with high sexual excitation and problematic sexual behavior who showed high approach/avoidance tendencies, reported higher symptoms of cybersex addiction. Analogous to substance dependencies, results suggest that both approach and avoidance tendencies might play a role in cybersex addiction. Moreover, an interaction with sensitivity toward sexual excitation and problematic sexual behavior could have an accumulating effect on the severity of subjective complaints in everyday life due to cybersex use. The findings provide further empirical evidence for similarities between cybersex addiction and substance dependencies. Such similarities could be retraced to a comparable neural processing of cybersex- and drug-related cues.

## Introduction

In the last decade it has been discussed to extent the concept of addiction from substance-related to non-substance-related behaviors, which are frequently referred to as behavioral addictions (Jović and Đinđić, 2011; Olsen, 2011; Wölfling et al., 2013). One domain of this field, which is receiving growing attention, is Internet addiction. Although diverse terminologies are used to describe this phenomenon (Cash et al., 2012; Starcevic, 2013; Koo and Kwon, 2014; Spada, 2014), the term Internet addiction seems to be dominant, because studies have shown widespread similarities to substance dependencies (Young, 1998; Griffiths, 2005; Brand et al., 2014b; Kuss et al., 2014a). For example, there is empirical evidence pointing toward comparabletrol, and withdrawal (Weinstein and Lejoyeux, 2010; Kuss et al., 2014b,c). On a theoretical level, several researchers argued to distinguish between generalized and specific forms of Internet addiction (Davis, 2001; Brand et al., 2014a; Montag et al., 2015). In the current study, we focus on cybersex addiction, which is referred to as a specific Internet addiction (Meerkerk et al., 2006; Young, 2008; Kuss and Griffiths, 2011). Until today, a consensual definition of cybersex addiction is missing. However, it is reasonable to rely on the proposed criteria of Internet Gaming Disorder (APA, 2013) since both can be considered as specific forms of Internet addiction (Young et al., 1999; Brand et al., 2014b). Thus, a working definition of cybersex addiction should include symptoms like loss of control, preoccupation, withdrawal, and continuous engagement in online sexual activities despite negative consequences. Additionally, cybersex addiction should not only be associated with pornography consumption but possibly to all cybersex activities mentioned by Döring (2009). Besides the consumption of pornography, these activities further include using online sex-shops and sexual education/information, searching sexual contacts as well as using services related to sex-work (Döring, 2009). Though, at least for men, pornography seems to be the most relevant cybersex activity (Short et al., 2011). Further, cybersex addiction is viewed to be different from hypersexuality (Kafka, 2010) or sex addiction (Reay et al., 2013) since for cybersex addiction only online sexual activities are taken into account which are not related to physical sexual intercourse in real life.

In the current study, we investigated possible links between tendencies to approach or avoid pornographic stimuli and tendencies toward cybersex addiction. Such mechanisms have been shown to be crucial for addictive behaviors (e.g., Wiers et al., 2009), while there is growing evidence to classify Internet addiction in analogy to substance dependencies (for review see Brand et al., 2014b). In the context of cybersex addiction, approach/avoidance tendencies can be interpreted as inclinations which can either promote (approach) or suppress (avoidance) cybersex use. Regarding alcohol dependency, Breiner et al. (1999, p.198) provided a theoretical framework which suggests that there can be “largely independent inclinations to approach and avoid drinking.” Consequently, individuals may not only show tendencies to approach but also to avoid alcohol-related stimuli. Recently, Schiebener et al. (2015) provided first empirical data suggesting the existence of a similar framework for cybersex addiction. They found a quadratic association between performance in a monitoring task that includes pornographic pictures and symptoms of cybersex addiction.

### Approach-Avoidance Tendencies in Substance Dependencies

According to Breiner et al. (1999), tendencies to approach or avoid addiction-related stimuli are connected with cue-reactivity and craving, which are frequently investigated in addiction literature (for review see Tiffany and Wray, 2012). Cue-reactivity represents subjective and physiological responses to addiction-related cues (Drummond, 2001). A consensual definition of craving is still missing (for review see Skinner and Aubin, 2010). Craving is mostly referred to as a subjectively experienced urge to consume a drug (Sayette et al., 2000), while other approaches argue to additionally assess non-subjective craving reactions with physiological measures of cue-reactivity (Ooteman et al., 2006) or behavioral inclinations toward drug use (Marlatt, 1985; Buydens-Branchey et al., 1997). Further, neurophysiological theories refer to adaptations in the mesolimbic dopaminergic pathway due to repeated drug use and argue that craving might also occur as an unconscious urge to consume a substance, which is called “wanting” (e.g., Robinson and Berridge, 1993, 2001, 2008). However, cue-reactivity and craving seem to be related concepts (Drummond, 2001), while there is enough evidence to neglect a one-dimensional definition of craving (Skinner and Aubin, 2010).

**FIGURE 1:**
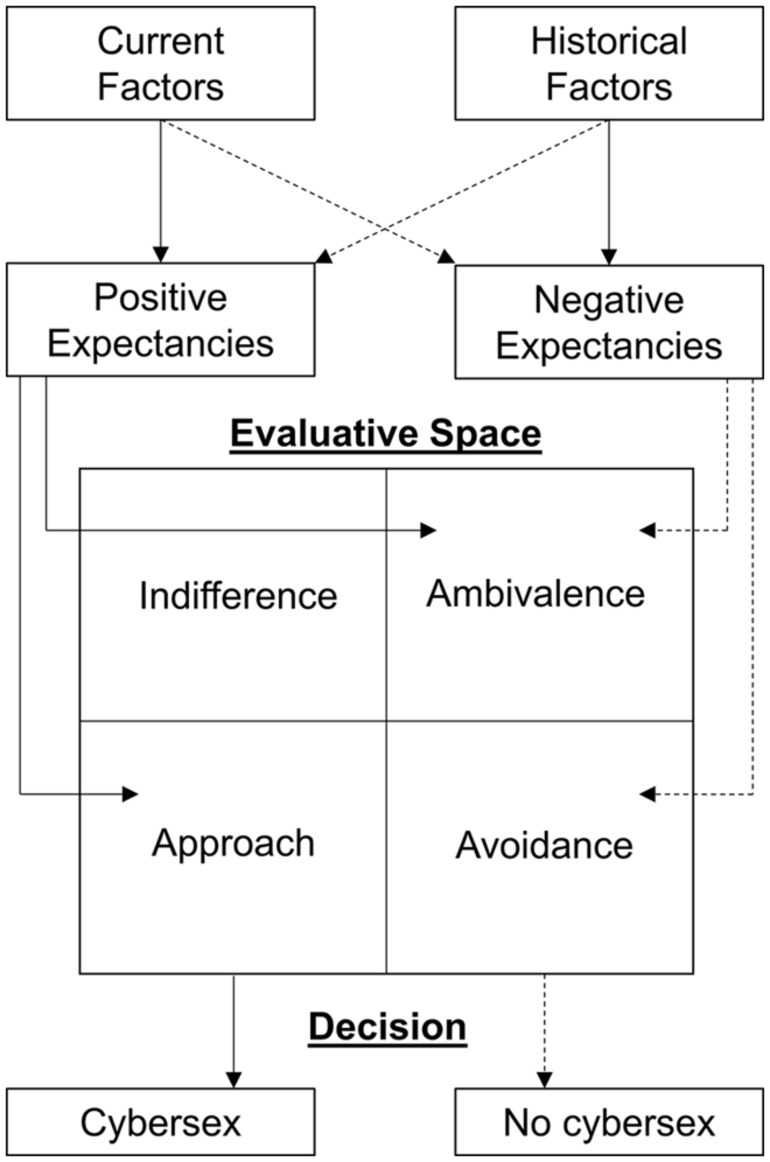
**Simplified overview of the approach/avoidance framework by Breiner et al. (1999) adapted to cybersex addiction.** Straight lines represent inclinations which may elicit tendencies promoting cybersex use while dashed lines rather embody tendencies to avoid cybersex use.

Striving for a differentiated definition of craving, Breiner et al. (1999) proposed a multi-dimensional model for alcohol dependency focusing on the role of an evaluative space in an addiction-related decision situation. The evaluative space can be divided into the states *approach*, *avoidance*, *ambivalence*, and *indifference*. *Approach* and *avoidance* are competing action-tendency states. Approach is supposed to cause alcohol consumption whereas avoidance represents an oppositional process in which the urge to consume alcohol is suppressed. Further, *ambivalence* and *indifference* can be described as ambiguous states, which might be entered if the inclinations of the action-tendency states are balanced. In this context, *ambivalence* represents a high and *indifference* a low intensity of ambiguity. Breiner et al. (1999) argue that the state entered in an addiction-related decision situation depends on either positive or negative expectancies toward drinking, which are further influenced by historical (e.g., psychological and physiological predispositions) as well as current factors (e.g., positive or negative incentives). Positive expectancies thereby promote the state *approach*, while negative expectancies are likely to cause *avoidance*. Regarding different aspects of craving, *approach* is synonymous with an irresistible “wanting” and thereby able to elicit an automatic response. Contrary, *avoidance* is supposed to be a subjectively experienced process. Consequently, the approach/avoidance framework is in line with dual-process models highlighting the role of automatic and controlled processes for the development and maintenance of addictive behaviors (e.g., Bechara, 2005; Wiers and Stacy, 2006). A simplified overview of the approach/avoidance framework by Breiner et al. (1999), which we have transferred to cybersex addiction, is summarized in **Figure 1**.

### Approach-Avoidance Tendencies in Cybersex Addiction

Based on the theoretical approach/avoidance framework by Breiner et al. (1999) and the suggested similarities between Internet addiction and substance dependencies it is plausible to assume comparable patterns in individuals with tendencies toward cybersex addiction. Regarding cue-reactivity and craving in cybersex addiction, studies already provided preliminary evidence for such similarities (Brand et al., 2011; Laier et al., 2013a). These studies indeed indicated that individuals with tendencies toward cybersex addiction showed both cue-reactivity and an increase of subjective craving when confronted with pornographic pictures. Further, sexual stimuli are known to induce neural activations which are similar to those induced by drug-related cues and are theoretically also able to promote adaptions in the mesolimbic dopaminergic pathway (Georgiadis and Kringelbach, 2012). Moreover, Laier and Brand (2014) recently proposed a theoretical framework for cybersex addiction which displays some similarities with the model by Breiner et al. (1999). For instance, the historical factors suggested by Breiner et al. (1999; e.g., person’s characteristics, past reinforcement, physiological reactivity) are in line with influences of specific predispositions toward sex as well as the suggested role of gratification proposed by Laier and Brand (2014). Further, Laier and Brand (2014) suggest a mediating role of cybersex use expectancies on the use of cybersex, which can be compared with the role of expectancies in the model by Breiner et al. (1999).

Regarding existing evidence for approach/avoidance tendencies in cybersex addiction, Schiebener et al. (2015) performed a study in which participants had to execute different tasks in a multitasking paradigm. These tasks were related to one of two picture sets, whereas the first picture set contained neutral and the second one contained pornographic pictures. Participants were instructed to execute all different tasks in equal measure, while they could switch autonomously between tasks and picture sets. The deviation from the optimal set balance was taken as dependent variable, indicating either a preference to work on the neutral or the pornographic set. Using this measure, the authors found a quadratic relationship between tendencies toward cybersex addiction and the deviation from set balance, which means that individuals with high tendencies toward cybersex addiction either preferred to work on the pornographic (approach) or on the neutral (avoidance) set. In contrast, participants with low tendencies toward cybersex addiction did not prefer to work more on one of the picture sets. Since the multitasking paradigm used by Schiebener et al. (2015) was not explicitly designed to measure tendencies to approach or avoid pornographic stimuli, it seems plausible to use a standard approach/avoidance paradigm in order to deeper investigate this phenomenon.

### Measuring Approach/Avoidance Tendencies

One way to assess tendencies to approach or avoid addiction-related stimuli is the Stimulus-Response-Compatibility Task (SRC; Field et al., 2006). During the SRC a manikin figure has to be moved toward and away from addiction-related cues in two separated blocks by using a standard keyboard. The difference between the mean reaction times (RTs) recorded in the two blocks is thereby supposed to reflect the relative inclination to either approach or avoid addiction-related cues. Several studies using the SRC showed stronger tendencies to approach than to avoid addiction-related stimuli in smokers (Mogg et al., 2003), regular cannabis users (Field et al., 2006), as well as heavy alcohol and cannabis users (Field et al., 2008; Cousijn et al., 2012). Regarding relationships between subjective craving and tendencies to approach or avoid addiction-related stimuli, outcomes have been inconsistent regarding whether these relationships may be linear or quadratic (Field et al., 2005a,b; Schoenmakers et al., 2008). As an extension of the SRC, Rinck and Becker (2007) introduced the Approach-Avoidance-Task (AAT), which includes physical movement to enhance effects of approaching and avoiding pictorial stimuli. By using a joystick, participants have to pull stimuli presented on a computer screen toward themselves (approach) or push them away (avoidance) from themselves. Originally, the AAT was designed to investigate fear-related behaviors (Rinck and Becker, 2007). Later, since competing tendencies to approach or avoid addictive behaviors are supposed to be essential in addiction-related decision situations (Breiner et al., 1999), modified versions of the AAT were used in studies regarding smoking (Wiers et al., 2013), a heavy use of cannabis (Cousijn et al., 2011, 2013) and alcohol dependency (e.g., Wiers et al., 2009; Sharbanee et al., 2013, 2014). In this context, most experimental studies found linear relationships between addictive behaviors and the tendency to approach addiction-related stimuli. However, in accordance with the dual-process models of addiction (Bechara, 2005; Wiers and Stacy, 2006), there is also empirical evidence for the assumption that addicted individuals might also show tendencies to avoid addiction-related stimuli, e.g., as a consequence of computerized avoidance training programs (Wiers et al., 2011; Eberl et al., 2013a,b). Moreover, Spruyt et al. (2013) found that abstaining alcohol-dependent individuals had, compared to matched controls, avoidance tendencies in an SRC, while relapse rates were positively associated with the strength of avoidance tendencies.

### Aims and Hypotheses

The aim of the current study is to investigate whether approach/avoidance tendencies might be mechanisms underlying cybersex addiction. While relying on the theoretical framework by Breiner et al. (1999) as well as the results provided by Schiebener et al. (2015), we expect to find that individuals with high tendencies toward cybersex addiction either show approach or avoidance tendencies toward pornographic stimuli. Additionally, low tendencies toward cybersex addiction should go along with balanced tendencies to approach or avoid pornographic stimuli. On an operationalized level, the relationship between approach/avoidance tendencies and cybersex addiction is supposed to be not linear but quadratic. Moreover, it is assumed that there will be neither a linear nor a quadratic relationship between tendencies to approach or avoid neutral stimuli and tendencies toward cybersex addiction. Further, since sensitivity toward sexual arousal as well as problematic sexual behavior were shown to promote the development and maintenance of cybersex addiction (Laier and Brand, 2014), we hypothesize that a combination of approach/avoidance tendencies toward pornographic pictures and a high problematic sexual behavior/sensitivity toward sexual excitation should have an accumulating effect on the severity of subjective complaints in everyday life due to the use of cybersex activities.

## Materials and Methods

### Participants

In the present study a total of 123 heterosexual male participants were examined (*M_age_* = 23.79 years, SD = 5.10). The mean age of first cybersex use was 15.61 (SD = 4.01) years. On average, participants used cybersex sites 3.66 (SD = 3.52) times per week, while spending *M_time_* = 22.25 (SD = 14.22) minutes per visit. Only participants of legal age (at least 18 years-old) were recruited. Recruitment was done through local advertisements at the University of Duisburg-Essen (Germany) and online platforms. It was stated in the advertisements that explicit pornographic material would be presented. Students could collect credits, non-student participants were paid €10 for participation. All participants gave written informed consent prior to the experiment and were debriefed at the end of the study. The study was approved by a local ethics committee.

### Measures

#### Pornographic Picture Rating

Before the AAT, participants watched and rated 50 pornographic pictures with respect to sexual arousal ranging from 1 (= *not sexually arousing*) to 5 (= *highly sexually arousing*). The stimulus set contained 10 different cybersex categories: heterosexual sex (vaginal sex, anal sex, cunnilingus, and fellatio), homosexual sex (anal and oral sex between two men, tribadism and oral sex between two women) as well as single masturbating men and women. Each category consisted of five pornographic pictures showing sexually explicit scenes without fetish relevant material. The internal consistency was very good (Cronbach’s α = 0.954). The same paradigm was used in several other studies, except that 100 pictures (10 per category) were used (Laier et al., 2013a,b, 2014).

Additionally, as described by Laier et al. (2013b), sexual arousal and the need to masturbate were measured before (*t1*) and after (*t2*) the pornographic picture rating on two horizontal sliders from 0 (= *not sexually aroused/no need to masturbate*) to 100 (= *very sexually aroused/great need to masturbate*). By subtracting *t1* from *t2* measurement, Δ-scores representing a relative increase or decrease of sexual arousal (craving Δ sexual arousal) and need to masturbate (craving Δ need to masturbate) were calculated and used as an operationalization of craving.

#### Approach-Avoidance-Task

Participants performed a modified version of the AAT (Rinck and Becker, 2007), in which pictures presented on a computer screen either had to be pulled toward (approach) or pushed away (avoidance) from their body with a joystick. Every single trial had to be manually started by the participant by pressing a button on the joystick, while the joystick had to be in the default position. Following a 500 ms inter-trial interval (ITI), a pictorial cue was presented. Due to the joystick movement, an implemented zooming feature increased (pull-movement) or decreased (push-movement) the size of the cue. In accordance to Rinck and Becker (2007), the joystick had to be moved ∼30° in one direction in order to terminate the trial. Further, a logarithmic growth function was used to increase or decrease cue size in order to allow participants to experience changes in cue size as immediate reactions to their joystick movements. All cues had an initial size of 700 × 500 pixel and were presented on a 15.6 inch screen. Due to moving the joystick ∼30° to one direction, the cue size changed to a maximum of 2100 × 1500 pixel (pull-movement), respectively a minimum of 233 × 166 pixel (push-movement). At the end of each trial, another 500 ms ITI was presented. The participants’ RTs were recorded in each trial. Similar to previous studies, the stimuli were separated into addiction-related and neutral cues (Wiers et al., 2009, 2013; Cousijn et al., 2011). As neutral cues, 40 pictures of the International Affective Picture System (IAPS; Lang et al., 2008) were used. Pictures showed one or two persons in neutral situations. As addiction-related cues we used 40 pornographic pictures out of four categories, which Laier et al. (2013a) identified as being sexually arousing for heterosexual men (heterosexual intercourse as vaginal sex and fellatio, homosexual intercourse between two women in the form of tribadism and oral sex). Additionally, five neutral and five pornographic pictures, which were not taken for the experimental trials, were used in the practice trials. Overall, the AAT and the pornographic picture rating used different pornographic cues.

During instruction, participants completed 30 practice trials, which were separated into four rounds (push, pull, porn-push/neutral-pull, porn-pull/neutral-push). After each round, participants were informed about the amount of correct reactions and could decide to repeat the round. The experimental trials were divided into four blocks with 80 trials each, resulting in a total of 320 trials. Every stimulus was presented once during one block in a semi-random order (maximally three stimuli of the same category were allowed to appear in a row). Participants were randomly assigned to one of two experimental conditions, which differed with respect to the instruction in the first block (porn-push/neutral-pull or porn-pull/neutral-push). In the following blocks the instruction was inverted. The experimental condition was counterbalanced across participants. By separating the type of instruction (direct vs. indirect), previous studies used different versions of the AAT. Versions with direct instructions (e.g., Rinck and Becker, 2007) included two stimulus categories, while indirect AATs (e.g., Wiers et al., 2009) used more than two stimulus categories and instructed participants to push or pull the joystick dependent on the picture format (horizontal vs. vertical). Thus, indirect AATs represent task-irrelevant designs, while direct AATs embody task-relevant paradigms. In this study, a task-relevant AAT was used, since a meta-analysis by Phaf et al. (2014) could not provide evidence for an advantage of task-irrelevant versions.

To analyze AAT data, median RT scores were calculated since medians are less vulnerable with respect to RT outliers than mean scores (Palfai, 2006; Wiers et al., 2009; Cousijn et al., 2013). RTs < 200 ms, > 2000 ms as well as RTs from false responses were discarded. An error rate >25% led to a complete exclusion from the data analysis. For each participant a compatibility effect score (Rinck and Becker, 2007) for both the pornographic (pornographic approach/avoidance score) and the neutral (neutral approach/avoidance score) stimulus category was calculated by subtracting the median pull from the median push RT (median RT push – median RT pull). According to Rinck and Becker (2007, p. 110), the compatibility effect score represents the “relative strength of approach and avoidance tendencies” while positive values indicate approach (median RT push > median RT pull) and negative values avoidance (median RT push < median RT pull) tendencies. The basic idea of these scores is that compatible trials (e.g., approach pornographic pictures) lead to faster RTs compared to incompatible trials (e.g., avoid pornographic pictures). Moreover, the pornographic approach/avoidance score is the main dependent variable, while the neutral approach/avoidance score represents a control variable, since approaching and avoiding neutral stimuli should not be connected to other dependent variables such as tendencies toward cybersex addiction.

Additionally, an overall effect score (overall RT score) was calculated by subtracting the median RT for all neutral stimuli from the median RT for all pornographic stimuli (median RT porn – median RT neutral). While the movement direction in specific trials is not taken into account for this measure, negative values indicate that participants were faster to respond to pornographic stimuli (median RT porn < median RT neutral), while positive values point toward slower RTs for pornographic stimuli (median RT porn > median RT neutral). Thus, the overall RT score is equivalent to the assessment of indirect attentional biases in substance use disorders (Field and Cox, 2008; Coskunpinar and Cyders, 2013; Field et al., 2014) than to measuring approach/avoidance tendencies with respect to stimulus type (pornographic vs. neutral). In analogy to substance dependency research, positive values of the overall RT score indicate the existence of an attentional bias toward pornographic pictures (slower RTs to pornographic compared to neutral stimuli). A general overview of all dependent variables of the AAT is summarized in **Table 1**. The AAT was programmed using Presentation®software (Version 16.5, www.neurobs.com).

**Table 1:** Calculation and interpretation of AAT scores.

	Pornographic approach/avoidance score	Neutral approach/avoidance score	Overall reaction time score
Construct	Relative strength of approach/avoidance tendencies toward pornographic pictures	Relative strength of approach/avoidance tendencies toward neutral pictures	Attentional bias toward pornographic pictures
Calculation	Median RT porn push – median RT porn pull	Median RT neutral push – median RT neutral pull	Median RT porn – median RT neutral
Interpretation	High values indicate a tendency to approach pornographic stimuli (faster RTs for pulling compared to pushing pornographic pictures) Low values indicate a tendency to avoid pornographic stimuli (slower RTs for pulling compared to pushing pornographic pictures)	High values indicate a tendency to approach neutral stimuli (faster RTs for pulling compared to pushing neutral pictures) Low values indicate a tendency to avoid neutral stimuli (slower RTs for pulling compared to pushing neutral pictures)	High values indicate an attentional bias toward pornographic pictures (slower RTs for pornographic compared to neutral pictures)
Reference	Rinck and Becker (2007)	Rinck and Becker (2007)	Field and Cox (2008)

#### Questionnaires

To assess tendencies toward cybersex addiction a short version of the Internet Addiction Test (s-IAT; Pawlikowski et al., 2013), modified for cybersex (s-IATsex; Laier et al., 2013a) was used. The s-IATsex consists of 12 items answered on a scale from 1 (= *never*) to 5 (= *very often*). The internal consistency of the s-IATsex in this study was good (Cronbach’s α = 0.846). It can be divided into the subscales *loss of control/time management* (s-IATsex time; e.g., “How often do you find that you stay on Internet sex sites longer than you intended?”) and *craving/social problems* (s-IATsex craving; e.g., “How often do you feel preoccupied with online sexual activities when off-line, or fantasize about being on Internetsex sites?”). Both s-IATsex time and s-IATsex craving have a possible range of 6–30.

Additionally, as a measure of general problematic sexual behavior, the Hypersexual Behavioral Inventory was used (HBI; Reid et al., 2011). The HBI contains 19 items rated on a scale between 1 (= *never*) and 5 (= *very often*) and can be separated into the subscales *loss of control* (e.g., “My sexual cravings and desires feel stronger than my self-discipline.”; Possible range: 8–40), *coping* (e.g., “I use sex to forget about the worries of daily life.”; Possible range: 7–35), and *consequences* (e.g., “My sexual behavior controls my life.”; Possible range: 4–20). In this study, the internal consistency of the HBI was good (Cronbach’s α = 0.885). Further, sensitivity toward sexual excitation was assessed by the Sexual Excitation Scale (SES; Carpenter et al., 2010), which consists of six items (e.g., “When I think someone sexually attractive wants to have sex with me, I quickly become sexually aroused.”). The internal consistency of the SES in this study was good (Cronbach’s α = 0.785). Compared to the version from Carpenter et al. (2010), the response format was inverted, which led to a scale from 1 (= *strongly disagree*) to 4 (= *strongly agree*), leading to an overall mean score of 6–24. At last, sociodemographic data as well as basic information with respect to pornography consumption were assessed.

The subscales s-IATsex craving and HBI loss of control will be used as dependent variables for testing the hypotheses since these scales assess subjective consequences of craving more specifically than the sum scores of the s-IATsex and the HBI. Thus, these scores are preferred for investigating the relationship between tendencies to approach or avoid pornographic stimuli and craving as it is suggested by Breiner et al. (1999). Further, high scores in the s-IATsex, the HBI and the SES represent tendencies toward pathological behavior patterns (e.g., high tendencies toward cybersex addiction, high loss of control with respect to sexual behaviors, high sexual excitation).

### Short- and Long-Term Measurements

The instruments used in the current study can be separated into short-term (pornographic picture rating, craving Δ sexual arousal/masturbation, AAT) and long-term measurements (s-IATsex, HBI, SES). In this context, short-term measurements refer to reactive (immediate) responses, which might be influenced by environmental factors, such as preceding cybersex consumption. In contrast, long-term measurements rather resemble individual characteristics, which are supposed to remain stable over a longer period of time.

### Statistical Analyses

Data analysis was done using IBM, SPSS Statistics Version 22.0. Relationships between two variables were analyzed with Pearson correlations. Differences between two variables were evaluated with one sample *t*-tests. Effect sizes are reported according to Cohen (1988) using Pearson’s *r* (*r* = 0.10, small; *r* = 0.30, medium; *r* = 0.50, large) and Cohen’s *d* (*d* = 0.20, small; *d* = 0.50, medium; *d* = 0.80, large). Quadratic relationships between two variables were evaluated using curve-linear regression analyses. Further, interactions between two variables as predictors of a single dependent variable were analyzed with hierarchical moderated regression analyses (all predictors centralized; Cohen et al., 2003). The significance level for all statistical tests was *p* = 0.05. Further, in order to check whether variables violated the assumption of normality, skewness, and kurtosis are reported in **Table 2**. According to West et al. (1995), skewness < | 2.00| and kurtosis < | 7.00| indicate that a variable is distributed normally. Here, all variables used for curve-linear and moderated regression analyses fulfilled these criteria (s-IATsex craving, HBI loss of control, SES, pornographic/neutral approach/avoidance score). However, in case other variables, which were used for further calculations, violated the assumption of normality, parametric tests were applied nonetheless, since it was shown that the parametric statistical methods are robust against this violation (Rasch and Guiard, 2004).

**Table 2:** Mean values of the s-IATsex, HBI, SES, pornographic picture rating and subjective ratings of sexual arousal as well as need to masturbate and AAT scores.

	Minimum	Maximum	*M*	SD	Skewness	Kurtosis
**s-IATsex^a^**	12	36	19.40	6.18	0.83	0.00
Loss of control/time management	6	22	9.96	3.95	1.02	0.37
Craving/social problems	6	19	9.44	3.07	0.91	0.36
**HBI^a^**	19	61	36.02	10.40	0.49	-0.51
Loss of control	8	27	14.39	5.12	0.75	-0.36
Coping	7	34	15.11	4.96	0.48	0.68
Consequences	4	15	6.52	2.60	1.39	1.61
**SES**^**b**^	9	24	16.55	2.98	-0.11	-0.37
**Pornographic picture rating**^**c**^
Heterosexual pictures	1.03	4.57	3.10	0.70	-0.43	-0.07
Homosexual pictures	1.00	1.67	1.04	0.11	3.34	12.51
**Sexual arousal**^**d**^
*t1*	0	80	6.85	13.37	3.15	11.26
*t2*	0	100	21.73	20.83	1.38	1.52
Craving Δ sexual arousal	-25	99	14.89	18.24	1.85	5.03
**Need to masturbate**^**e**^
*t1*	0	63	4.33	9.15	3.65	16.51
*t2*	0	61	11.68	14.52	1.64	2.20
Craving Δ masturbation	-7	60	7.36	11.18	2.26	6.23
**Approach-Avoidance-Task**^**f**^
Pornographic approach/avoidance score	-201	235	-1.09	72.64	0.42	1.19
Neutral approach/avoidance score	-219	67	-56.91	55.03	-0.23	-0.06
Overall reaction time score	-133	101	-37.79	42.74	0.73	1.21

^a^Scale from 1 = *never* to 5 = *very often*. ^b^Inverted scale from 1 = *strongly disagree* to 4 = *strongly agree*. High SES-scores represent high sensitivity for sexual excitation. ^c^Scale from 1 = *not sexually arousing* to 5 = *highly sexually arousing*. ^d^Scale from 0 = *not sexually aroused* to 100 = *very sexually aroused*. ^e^Scale from 0 = *no need to masturbate* to 100 = *very great need to masturbate*. ^f^Positive approach/avoidance scores reflect approach, while negative values resemble avoidance tendencies.

## Results

### Pornographic Picture Rating

A one-sample *t*-test was calculated to compare the ratings for heterosexual and homosexual pictures, *t*(122) = 32.79; *p* < 0.001; *d* = 4.11, indicating that heterosexual pictures were rated as significantly more sexually arousing. Regarding the evaluation of sexual arousal and the need to masturbate before (*t1*) and after (*t2*) the pornographic picture rating, two *t*-tests for dependent samples revealed higher subjective sexual arousal, *t*(122) = -9.05; *p* = 0.001; *d_z_* = 0.85, and a higher need to masturbate, *t*(122) = -7.30; *p* < 0.001; *d_z_* = 0.61, at *t2* compared to *t1* (for mean values see **Table 2**). These results indicate that due to watching pornographic pictures, participants experienced a state of being sexually aroused before starting the AAT. This is of particular importance since sexual arousal and the need to masturbate are operationalized as craving measures, which are supposed to be linked to tendencies to approach or avoid pornographic stimuli.

### Approach-Avoidance-Task

Descriptively, the pornographic approach/avoidance score (*M* = -1.09, SD = 72.64) and the neutral approach/avoidance score (*M* = -56.91, SD = 55.03) had negative mean values. These results indicate a mean tendency for avoiding both pornographic and neutral stimuli in the AAT, while this effect was stronger for neutral stimuli, *t*(122) = 8.52; *p* < 0.001; *d* = 0.87. In contrast, the overall RT score (*M* = -37.79, SD = 42.74) had a negative mean value, which indicates that participants on average had no attentional bias toward pornographic stimuli (such an attentional bias would be reflected by slower RTs for pornographic pictures and therefore a positive mean overall RT, which is not the case, since we observed faster RTs for pornographic compared to neutral stimuli).

Correlations between the AAT scores and selected variables are summarized in **Table 3**. Regarding the pornographic and the neutral approach/avoidance score there were no significant correlations with other measures. However, the overall RT score correlated significantly with sensitivity toward sexual excitation, the HBI loss of control scale as well as the craving Δ sexual arousal and the craving Δ need to masturbate score.

**Table 3:** Bivariate correlations between the AAT scores and selected variables.

	1	2	3	4	5	6	7	8	9	10	11	12
(1) Pornographic approach/avoidance score^a^	–											
(2) Neutral approach/avoidance score^a^	0.013	–										
(3) Overall reaction time score^b^	0.031	-0.224^∗^	–									
(4) s-IATsex sum	-0.126	0.074	0.164	–								
(5) s-IATsex time	-0.102	-0.015	0.124	0.909^∗∗^	–							
(6) s-IATsex craving	-0.122	0.168	0.172	0.845^∗∗^	0.545^∗∗^	–						
(7) HBI sum	0.029	0.024	0.211^∗^	0.568^∗∗^	0.498^∗∗^	0.503^∗∗^	–					
(8) HBI loss of control	0.019	0.040	0.212^∗^	0.473^∗∗^	0.375^∗∗^	0.471^∗∗^	0.827^∗∗^	–				
(9) HBI coping	0.008	-0.042	0.162	0.459^∗∗^	0.429^∗∗^	0.373^∗∗^	0.821^∗∗^	0.403^∗∗^	–			
(10) HBI consequences	0.064	0.098	0.117	0.464^∗∗^	0.436^∗∗^	0.374^∗∗^	0.806^∗∗^	0.567^∗∗^	0.580^∗∗^	–		
(11) SES^c^	0.130	0.172	0.261^∗∗^	0.424^∗∗^	0.378^∗∗^	0.367^∗∗^	0.404^∗∗^	0.319^∗∗^	0.310^∗∗^	0.395^∗∗^	–	
(12) Craving Δ sexual arousal	-0.065	-0.131	0.250^∗∗^	0.164	0.109	0.189^∗^	0.298^∗∗^	0.203^∗^	0.322^∗∗^	0.179^∗^	0.194^∗^	–
(13) Craving Δ masturbation	-0.117	-0.147	0.392^∗∗^	0.179^∗^	0.134	0.187^∗^	0.203^∗^	0.081	0.316^∗∗^	0.049	0.169	0.734^∗∗^

^∗^*p* < 0.05, ^∗∗^*p* < 0.01. ^a^Positive approach/avoidance scores reflect approach, while negative values resemble avoidance tendencies. ^b^Positive values indicate an attentional bias toward pornographic pictures. ^c^Inverted scale. High SES-scores represent high sensitivity for sexual excitation.

#### Curve-Linear Regression Analysis

**FIGURE 2:**
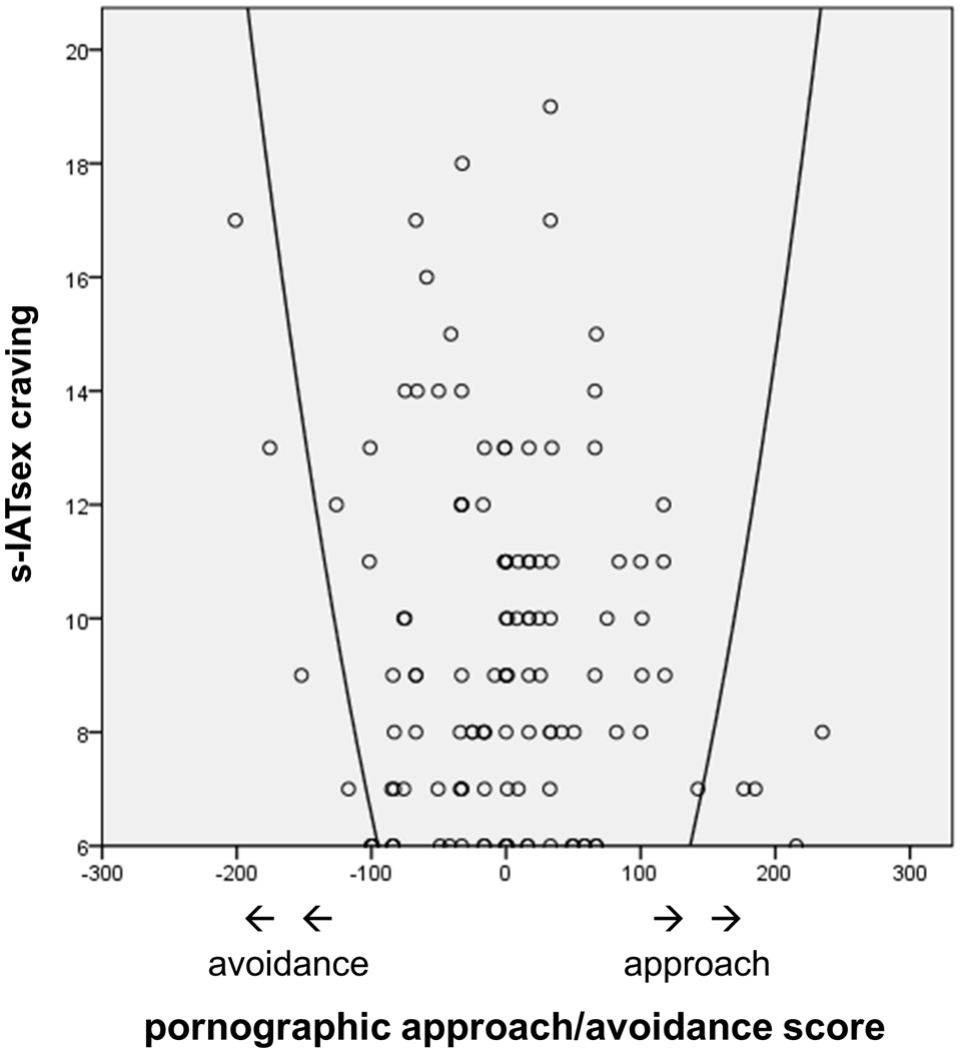
**Relationship between the compatibility effect score for pornographic pictures (pornographic approach/avoidance score) and the s-IATsex factor craving**.

To test whether the relationship between the pornographic approach/avoidance score and the s-IATsex factor craving was not linear but quadratic, a curve-linear regression analysis was calculated. In a first step the pornographic approach/avoidance score was entered but did not significantly explain s-IATsex craving variance, *R*^2^ = 0.003, *F*(1,122) = 0.33, *p* = 0.567, which indicates that no linear relationship between the two variables exists in the data. In a second step the squared pornographic approach/avoidance score was included, which led to a significant explanation of 23.7% of the s-IATsex craving variance, Δ*R*^2^ = 0.234, *F*(1,122) = 18.80, *p* < 0.001. This estimated curve (see **Figure 2**) indicates that individuals with a high s-IATsex craving tended to either show approach (positive approach/avoidance values) or avoidance (negative approach/avoidance values) inclinations with respect to pornographic stimuli. Further regression values are summarized in **Table 4**.

**Table 4:** Values of the curve-linear regression analysis with the s-IATsex factor craving as dependent variable.

		β	*T*	*P*	Δ*R*^2^
**Dependent variable: s-IATsex craving**
Step 1	Pornographic approach/avoidance score	-0.141	-1.74	0.084	0.003
Step 2	(Pornographic approach/avoidance score)^2^	0.492	6.10	<0.001	0.234

As a manipulation check, a second analysis was calculated to investigate the relationship between the s-IATsex craving and the neutral approach/avoidance score. Here, no significant quadratic relationship could be found (*p* = 0.239).

#### Moderated Regression Analyses

To investigate the relationship between sensitivity toward sexual excitation (SES), tendencies to approach or avoid pornographic stimuli (pornographic approach/avoidance score), and tendencies toward cybersex addiction, a hierarchical moderated regression analysis with the s-IATsex factor craving as dependent variable was calculated (all variables centralized; Cohen et al., 2003). In the first step, the *SES* explained 13.5% of the s-IATsex craving variance, *F*(1,121) = 18.83, *p* < 0.001. In the second step, the *pornographic approach/avoidance score* led to a significant increase of variance explanation, Δ*R^2^* = 0.029, Δ*F*(2,120) = 4.19, *p* = 0.043. In the third step, the interaction of the *SES* and the *pornographic approach/avoidance score* led to a significant increase of variance explanation, Δ*R^2^* = 0.044, Δ*F*(3,119) = 6.62, *p* = 0.011. Overall, the regression model was significant and explained 20.8% variance of the s-IATsex craving, *F*(3,122) = 10.41, *p* < 0.001.

**FIGURE 3:**
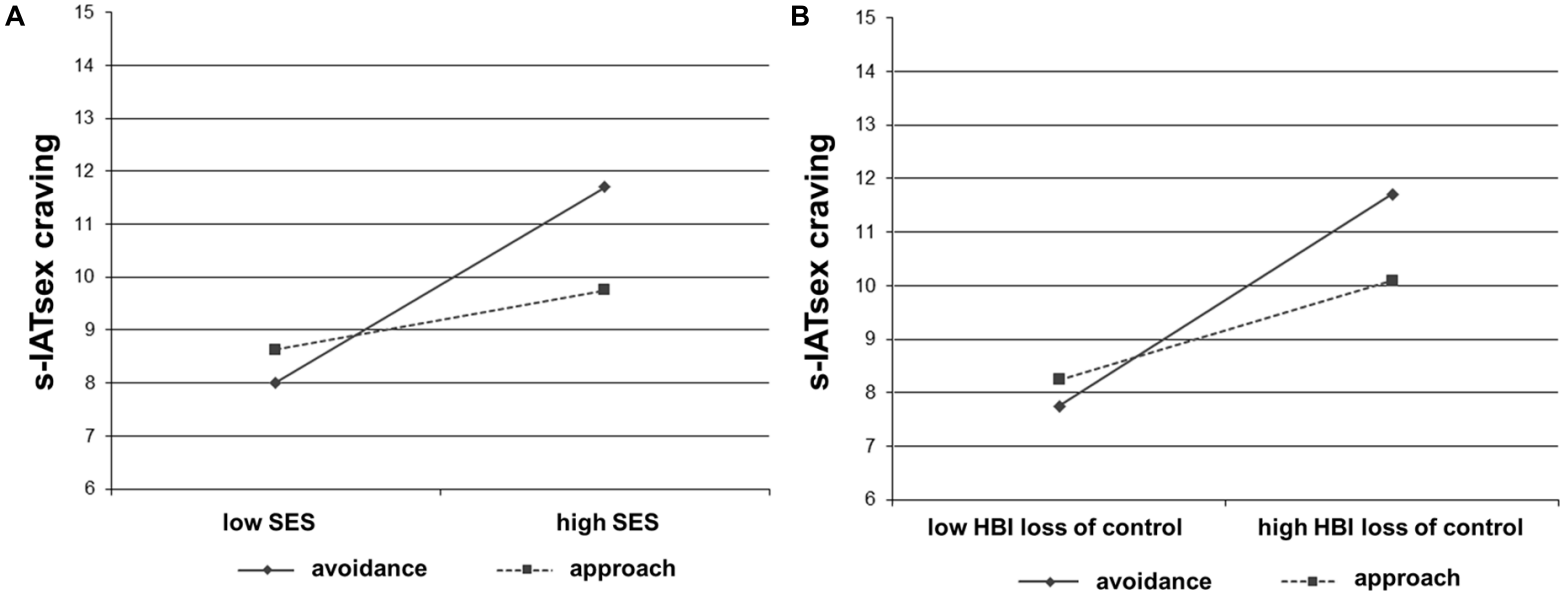
**Graphical illustration of simple slopes with respect to the interactions between the compatibility effect for pornographic pictures (pornographic approach/avoidance score) and **(A)** the sensitivity toward sexual excitation (SES) as well as **(B)** problems controlling sexual behavior (HBI loss of control)**.

To investigate the observed moderation effect in more detail, simple slopes were analyzed (see **Figure 3A**). The slope of the regression line representing *approach tendencies* (1 standard deviation above the mean) was not significantly different from zero, *t* = 1.71, *p* = 0.090. In contrast, the slope of the regression line representing *avoidance tendencies* (1 standard deviation below the mean) was significantly different from zero, *t* = 5.50, *p* < 0.001, indicating that a *high SES*, accompanied by *avoidance tendencies* resulted in a high s-IATsex craving score. When using tendencies to approach or avoid neutral stimuli (neutral approach/avoidance score) as moderator, no significant interaction could be found (*p* = 0.196).

A second model was calculated to investigate the relationship between the control component of problematic sexual behavior (HBI loss of control), tendencies to approach or avoid pornographic stimuli (pornographic approach/avoidance score), and tendencies toward craving components in cybersex addiction. In the first step, the *HBI loss of control* explained 22.2% of the s-IATsex craving variance, *F*(1,121) = 34.52, *p* < 0.001. In the second step, the *pornographic approach/avoidance score* did not lead to a significant increase of variance explanation, Δ*R^2^* = 0.017, Δ*F*(2,120) = 2.70, *p* = 0.103. In the third step, the interaction of the *HBI loss of control* and the *pornographic approach/avoidance score* led to a significant increase of variance explanation, Δ*R^2^* = 0.037, Δ*F*(3,119) = 6.02, *p* = 0.016. Overall, the regression model was significant while explaining 25.7% variance of the s-IATsex craving, *F*(3,122) = 15.10, *p* < 0.001. Further values for both moderated regression analyses are summarized in **Table 5**.

**Table 5:** Values of the moderated regression analyses with s-IATsex factor craving as dependent variable.

		β	*T*	*P*	*ΔR^2^*
**Model 1: Moderated regression analysis**	
Main effects	SES^a^	0.393	4.78	<0.001	0.135
	Pornographic approach/avoidance score	-0.108	-1.26	0.210	0.029
Interaction	SES × pornographic approach/avoidance score	-0.220	-2.57	0.011	0.044
**Model 2: Moderated regression analysis**	
Main effects	HBI loss of control	0.474	6.07	<0.001	0.222
	Pornographic approach/avoidance score	-0.092	-1.15	0.251	0.017
Interaction	HBI loss of control × pornographic approach/avoidance score	-0.195	-2.45	0.016	0.037
**Model 3: Linear regression analysis**	
Main effects	HBI loss of control	0.390	4.82	<0.001	0.222
	SES	0.264	3.23	0.002	0.052
	pornographic approach/avoidance score	-0.163	-2.21	0.037	0.026

^a^Inverted scale. High SES-scores represent high sensitivity for sexual excitation.

Similar to the first model, the simple slopes were analyzed (see **Figure 3B**). The slope of the regression line representing *approach tendencies* (1 standard deviation above the mean) was significantly different from zero, *t* = 2.85, *p* = 0.005. The slope of the regression line representing *avoidance tendencies* (1 standard deviation below the mean) was also significantly different from zero, *t* = 6.14, *p* < 0.001, indicating that both *approach* and *avoidance* toward pornographic pictures, accompanied by a *high HBI loss of control* resulted in a high s-IATsex craving score. Similar to the first moderated regression analysis, using tendencies to approach or avoid neutral stimuli (neutral approach/avoidance score) as moderator showed no significant interaction (*p* = 0.166).

Additionally, to investigate whether the HBI loss of control scale, the SES, and the pornographic approach/avoidance score have an accumulating effect on tendencies toward cybersex addiction, a linear regression analysis with the s-IATsex factor craving as dependent variable was calculated. In the first step, the *HBI loss of control* explained 22.2% of the s-IATsex craving variance, *F*(1,121) = 34.52, *p* < 0.001. In the second step, the *SES* led to a significant increase of variance explanation, Δ*R^2^* = 0.052, Δ*F*(2,120) = 2.63, *p* = 0.004. In the third step, the *pornographic approach/avoidance score* led to a significant increase of variance explanation, Δ*R^2^* = 0.024, Δ*F*(3,119) = 4.47, *p* = 0.037. Overall, the regression model was significant and explained 30.1% variance of the s-IATsex craving, *F*(3,122) = 17.04, *p* < 0.001. Further regression values are summarized in **Table 5**.

### Relationships between Actual Cybersex use and Addiction-Related Measurements

In order to investigate possible relationships between actual cybersex use and measurements related to cybersex addiction, several additional correlations were calculated. There were positive relationships between the s-IATsex factor craving and both the frequency of weekly cybersex use (*r* = 0.227, *p* = 0.011) and the average time spent on cybersex sites during one visit (*r* = 0.198, *p* = 0.028). However, no significant relationships could be found between the frequency of weekly cybersex use and HBI loss of control (*r* = 0.136, *p* = 0.133), SES (*r* = 0.119, *p* = 0.190) as well as craving Δ sexual arousal/masturbation and AAT scores (all *ps* > 0.400). Similarly, there were no significant relationships between the average time spent on cybersex sites during one visit and HBI loss of control (*r* = 0.025, *p* = 0.781), SES (*r* = 0.161, *p* = 0.076) as well as craving Δ sexual arousal/masturbation and AAT scores (all *ps* > 0.500).

## Discussion

The main result of this study is that tendencies toward cybersex addiction seem to be related to approach/avoidance tendencies. First, individuals who reported higher symptoms of cybersex addiction tended to either approach or avoid pornographic pictures, while this was not the case for neutral stimuli. Second, we found that sensitivity toward sexual arousal as well as problematic sexual behavior interacted with approach/avoidance tendencies toward pornographic pictures, leading to an accumulating effect on tendencies toward cybersex addiction. Again, no significant interactions were found for approach/avoidance tendencies toward neutral stimuli.

The results of this study preliminary indicate that approach/avoidance tendencies might be connected to excessive cybersex use and potentially to cybersex addiction. This is also in line with the data provided by Schiebener et al. (2015). Further, our findings fit well into the cybersex addiction model proposed by Laier and Brand (2014), because we found that the existence of specific predispositions indicated an increased severity of cybersex addiction symptoms while not being dependent on tendencies to approach or avoid pornographic stimuli to have an influencing effect. Moreover, while providing preliminary evidence regarding a quadratic relationship between symptoms of cybersex addiction and approach/avoidance tendencies, the results are in line with the proposed evaluative space by Breiner et al. (1999), which suggests that not only approach, but also avoidance can be shown by addicted individuals.

Regarding the interactions between specific predispositions toward cybersex and approach/avoidance tendencies, it is interesting to note that problematic sexual behavior led, accompanied by either approach or avoidance tendencies, to high subjective symptoms of cybersex addiction. Contrary, the interaction between sensitivity toward sexual arousal and approach/avoidance tendencies only showed a significant effect for avoidance tendencies. This finding might be explained by referring to Bechara (2005), who stated that addictive behaviors are affected by two separate neural systems: an impulsive (amygdala) system, reacting to immediate reward and punishment, and a reflected (prefrontal cortex) system, coding expectations of long-term consequences. Within functional behavior it is assumed that the impulsive system is controlled by the reflective system, while in addictive behaviors a hyperactive impulsive system may override the reflective system due to drug-related neuroadaptations (see Robinson and Berridge, 1993, 2001, 2008). Regarding tendencies to approach or avoid pornographic stimuli, it is likely that a domination of the impulsive system could induce tendencies to approach, while the reflective system could promote tendencies to avoid pornographic stimuli (Wiers and Stacy, 2006). Based on these theories, our findings could be explained as follows: it is plausible to assume that problematic sexual behavior is able to enhance the development of neuroadaptations, which could be responsible for impulsive approach tendencies since it has been shown that sex- and drug-related cues are similarly processed (see Georgiadis and Kringelbach, 2012). In contrast, it is unlikely that such neuroadaptations have been developed due to a high sensitivity toward sexual excitation, because this construct is rather related to a person’s specific characteristics. This leads to the assumption that a high sensitivity toward sexual excitation should not increase the probability of a tendency to approach pornographic stimuli in addicted individuals, while this should be the case for a highly problematic sexual behavior. However, if an urge to approach addiction-related stimuli can be suppressed, e.g., because such behaviors have been trained, avoidance tendencies can be seen as consequences of a controlled process. Subsequently, training effects could lead to a particular control of the reflective system over a hyperactive impulsive system, although dysfunctional neuroadaptations have been built. Additionally, it seems plausible to assume that individuals who report indicators of problematic sexual behavior and a high sensitivity toward sexual excitation could be more likely to already have experienced negative consequences in everyday life due to their sexual behavior. Following, the existence of these specific predispositions could also increase the awareness of a potentially problematic cybersex use. Thus, such individuals could have stronger inclinations to avoid pornographic stimuli due to a controlled processing, even though avoidance reactions have not been trained explicitly.

Thinking further, cybersex use characteristics such as the frequency of weekly cybersex use and the average time spent on cybersex sites during one visit were not connected to immediate measurements related to cybersex addiction like subjective craving or the dependent variables of the AAT. Thus, these results facilitate the assumption that the observed approach/avoidance tendencies could be derived from neural sensitizations due to a long-term exposure of cybersex-related cues. Moreover, actual cybersex use might be connected with the maintenance of an addictive use of cybersex, while our results suggest that the AAT rather measures effects which might be connected to a dysfunctional cybersex use, carried out over a longer period of time. However, further empirical evidence is needed to evaluate whether the AAT is a short-term or a long-term measurement.

Another side result of this study is that problematic sexual behavior, a high sensitivity toward sexual excitation, and high craving scores were positively associated with the overall RT score, which means that these variables correlated with slower RTs in pornographic compared to neutral trials. This finding is consistent with results from studies investigating attentional biases in addictive behaviors (for review see Field and Cox, 2008). Thereby, it is assumed that slower RTs to addiction-related stimuli can be observed because such stimuli capture the attention of addicted individuals. Of course, the AAT is no standardized paradigm to measure attentional bias, but these results at least point toward a possible importance of this phenomenon in cybersex addiction and could be investigated in upcoming studies.

### Future Directives

Future studies could further aim on expanding the focus of interest by including positive and negative expectancies as possible predictors for approach/avoidance tendencies in analogy to the proposed framework by Breiner et al. (1999). Thus, positive expectancies are supposed to promote inclinations to approach addictive behaviors, while negative expectancies could suppress such urges and lead to avoidance reactions. In the context of cybersex addiction, cybersex use expectancies could have a similar influence on approach/avoidance tendencies since it was already shown that Internet use expectancies are connected with Internet addiction (Brand et al., 2014a). Besides the existence of competing approach/avoidance inclinations, such expectancies could thereby explain which inclinations might be dominant in an addiction-related decision situation.

Moreover, it could be beneficial to investigate if competing neural networks are involved in approach/avoidance reactions. In this context, studies already showed parallels to the dual-process model by Bechara (2005) since different neural networks were preliminary shown for approach (nucleus accumbens, medial prefrontal cortex) and avoidance (amygdala, dorsolateral prefrontal cortex) in alcohol addicted individuals (Ernst et al., 2012; Wiers et al., 2014b). By strengthening this finding, Schlund et al. (2011) reported balanced activations of these networks for approach/avoidance behavior in healthy individuals. Moreover, it could be shown that cognitive bias modification programs reduced approach/avoidance related activations in the medial prefrontal cortex and in the amygdala (Wiers et al., 2014a, 2015). Based on these results, it seems plausible to assume that the AAT is able to measure both approach- and avoidance-biases. Consequently, future studies should address the investigation of neural correlates connected with approach/avoidance tendencies in cybersex addiction in order to strengthen the current studies’ findings. Moreover, both substance dependency and cybersex addiction research could benefit from the application of sophisticated analyses methods (e.g., bin-analyses). Thus, such methods could provide more evidence for the assumption that the AAT assesses both approach- and avoidance-biases.

Thinking further, previous studies mostly investigated linear relationships between approach/avoidance tendencies and addiction-related measurements, whereas such an approach might not cover the complexity of addictive behaviors. However, the most prominent belief seems to be that only approach-biases are connected to the development and maintenance of addictive behaviors, although this assumption is not entirely supported by existing findings. For instance, some studies reported approach-biases in individuals with problematic substance use (e.g., Cousijn et al., 2012), whereas avoidance tendencies were found in abstinent or treatment-seeking subjects (Eberl et al., 2013a). Moreover, Wiers et al. (2013) found approach-biases in smokers, but not in ex-smokers. Further, relationships between approach/avoidance tendencies and addiction-related measurements such as subjective craving or relapse rates are inconsistent since both positive (e.g., Mogg et al., 2005) as well as negative associations (e.g., Cousijn et al., 2011; Spruyt et al., 2013) have been reported. Therefore, it seems plausible to assume that not only approach, but also avoidance tendencies might be important factors within addictive behaviors. Thus, curve-linear regression analyses, which allow the analysis of both inclinations in a single model, might not only be beneficial for investigating cybersex addiction, but also for studying approach/avoidance tendencies in other behavioral addictions or substance dependencies.

At last, it might be useful to investigate to which extent approach/avoidance tendencies influence the development and maintenance of cybersex addiction. Here, longitudinal study designs could be beneficial. Moreover, such an approach seems plausible since the results of the current study suggested the AAT to measure effects due to long-term cybersex use, while more research is necessary to justify this assumption.

### Limitations

First of all, it has to be noted that the curve-linear regression analysis used to test the assumed quadratic relationship between approach/avoidance tendencies and craving-related symptoms of cybersex addiction may rather be considered as an exploratory method. Moreover, the results are not emphasizing a perfect quadratic relationship. Therefore, the findings have to be interpreted with caution and need to be replicated. Nonetheless, these results at least point toward a non-linearity of the relationship between approach/avoidance tendencies and cybersex addiction. Since we only included heterosexual male participants, our results can hardly be generalized to women or homosexual individuals. Further, the majority of the sample consisted of regular cybersex users, while a minority reported subjective symptoms in everyday life due to their cybersex use. Although, the investigation of disorders with analog samples offers many benefits (Abramowitz et al., 2014), our findings cannot be entirely transferred to a clinical population since none of the participants were diagnosed as being addicted to cybersex. Therefore, future studies could benefit from investigating individuals in a clinical setting, though it has to be noted that missing diagnostic criteria can make it difficult to compare a cybersex-addicted patient group with a control group in the classical way. However, such an approach could be useful because the AAT could also be used for cognitive bias modification training (Wiers et al., 2011) in cybersex addiction treatment.

## Conclusion

The results of this study preliminary suggest that approach/avoidance tendencies might be mechanisms which are connected with cybersex addiction. More specific, it was shown that individuals with tendencies toward cybersex addiction revealed both approach and avoidance tendencies, which is in accordance with theories from substance dependency research (Breiner et al., 1999; Wiers et al., 2007). In combination with the results presented by Schiebener et al. (2015), there is accumulating evidence for the assumption that both tendencies to approach or avoid pornographic stimuli can be shown by individuals with tendencies toward cybersex addiction. Consequently, the results need to be discussed with their relevance for analogies between cybersex addiction and substance dependencies.

## Conflict of Interest Statement

The authors declare that the research was conducted in the absence of any commercial or financial relationships that could be construed as a potential conflict of interest.
